# Complete Overlay Denture for Pedodontic Patient with Severe Dentinogenesis Imperfecta

**DOI:** 10.5005/jp-journals-10005-1472

**Published:** 2017-02-27

**Authors:** Gibi Syriac, Elizabeth Joseph, Suresh Rupesh, Josey Mathew

**Affiliations:** 1 Reader,Department of Pedodontics, Pushpagiri College of Dental Sciences, Tiruvalla, Kerala, India; 2 Professor and Head, Department of Pedodontics, Pushpagiri College of Dental Sciences, Tiruvalla, Kerala, India; 3 Associate Professor, Department of Pedodontics, Pushpagiri College of Dental Sciences, Tiruvalla, Kerala, India; 4 Professor, Department of Conservative Dentistry and Endodontics Pushpagiri College of Dental Sciences, Tiruvalla, Kerala, India

**Keywords:** Dentinogenesis imperfecta, Hereditary opalescent dentin, Overlay denture, Pediatric prosthodontics.

## Abstract

Dentinogenesis imperfecta (DI) is a hereditary condition that may affect both primary and permanent dentition and is characterized by abnormal dentin formation. The teeth may be discolored with chipping of enamel and, in untreated cases, the entire dentition may wear off to the gingiva. This may lead to the formation of abscesses, tooth mobility, and early loss of teeth. In the Indian population, DI is found to have an incidence of 0.09%. Treatment of DI should aim to remove infection, if any, from the oral cavity; restore form, function, and esthetics; and protect posterior teeth from wear for maintaining the occlusal vertical dimension. Treatment strategies should be selected based on the presenting complaint of the patient, patient’s age, and severity of the problem. This case report presents the management of severe DI with tooth worn off until gingival level in a very young patient using complete overlay denture, which has not been reported earlier.

**How to cite this article:** Syriac G, Joseph E, Rupesh S, Mathew J. Complete Overlay Denture for Pedodontic Patient with Severe Dentinogenesis Imperfecta. Int J Clin Pediatr Dent 2017;10(4):394-398.

## INTRODUCTION

Dentinogenesis imperfecta, also known as Capdepont teeth, hereditary opalescent dentin, and hereditary brown teeth, is a hereditary condition that affects both primary and permanent dentition characterized by abnormality in dentin formation. This condition was first described by Barret in 1882.^1^ Robert and Schour coined the term “DI” in 1939.^2^ Skillen, Finn, and Hodges coined the term “hereditary opalescent dentin.”^1^

The DI has an incidence of 1 in 8,000 births in the United States.^3^ In India, studies have found that 0.09% of the population is affected with DI.^4^

There are three types of DI as proposed by Shields et al in 1973. Type I DI is associated with osteogenesis imperfecta. Type II DI is not associated with osteogenesis imperfecta. Type III is rare and found only in Brandywine population of Maryland in the United States.^5^

Current evidences show that DI and osteogenesis imperfecta are two separate entities not related to each other. Hence, a revised classification is proposed— DI I and DI without osteogenesis imperfecta, corresponding to DI type II of Shields classification. DI II; Brandywine type, corresponding to DI type III of Shields classification. There is no substitute for Shields DI type I in this revised classification.^6^

The DI is a localized mesodermal dysplasia that affects both primary and permanent dentition. The inheritance of DI is in a simple autosomal dominant mode with high penetrance and low mutation rate.^1^

DI types II, III, and dentin dysplasia type II are caused because of mutations in the dentin sialophosphoprotein (DSPP) gene. So, these are not separate, but are allelic with differing severity. Several other candidate genes for nonsyndromic hereditary dentin defects may be present, but only mutations in the DSPP gene have been identified to date.^7^

The DSPP gene located on chromosome 4q21 encodes the major noncollagenous protein in the dentin matrix. The DSPP is cleaved by proteases into three major proteins: Dentin sialoprotein, dentin glycoprotein, and dentin phosphoprotein. Mutant DSPP gene results in reduction of DSPP and/or improper mineralization resulting in defective dentin mineralization.^8^ Alternatively, the mutant DSPP gene accumulates in the odontoblast, resulting in cellular damage and influencing protein processing and/or transporting system during rapid dentin matrix formation.

Clinically, in patients with DI, the primary and permanent dentition may show teeth with amber, gray, yellow, brown, purple, or bluish translucent discoloration. The severity of discoloration and extent of enamel fracture are highly variable, even within the same family. In untreated cases, the entire dentition may wear off to the gingiva. Abscesses, tooth mobility, and early loss of teeth may be seen. The tooth enamel may be chipped off with dentin exposed; the exposed dentin may be sclerosed with hard glassy appearance and, due to sclerosis, patients rarely complain of sensitivity.^6,9^

In radiographs, teeth present with bulbous crowns with marked cervical constriction, sharp conical roots with apical constrictions, or sometimes rootless teeth. In the initial stages of DI, pulp chamber of teeth appears abnormally wide resembling “shell teeth,” but they undergo progressive obliteration. Pulp stones may be seen. There may be numerous periapical radiolucencies in noncarious teeth.^6^

Histologically, in a normal tooth, dentinoenamel junction (DEJ) is scalloped, and this scalloping at the DEJ develops mechanical interlocking. In DI, this scalloping is absent resulting in no interlocking of enamel and dentin. This leads to early loss of enamel, and the exposed dentin may undergo severe and rapid attrition. The mantle dentin may be normal, but the dentinal tubules of the circumferential dentin are coarse and branched and total number of dentinal tubules is less. The atubular dentin shows reduced mineralization with reduced number of odontoblasts. Pulpal inclusions and increased interglobular dentin are also frequently seen.^3,6^

Chemical and physical analysis of teeth affected with DI shows that the water content is increased to as much as 60% above normal, and the inorganic content is less than that of normal dentin. Density, X-ray absorption, and hardness of dentin are also low and dentinal microhard-ness closely approximates that of cementum, resulting in the rapid attrition of teeth.^6^

Conditions with similar clinical or radiological features included in the differential diagnosis of DI are

- Hypocalcified form of amelogenesis imperfecta that leads to loss of enamel and exposure of underlying dentin.
- Intrinsic discolorations, e.g., congenital erythropoi-etic porphyria, rhesus incompatibility, tetracycline staining.
- Conditions that cause early teeth loss, e.g., vitamin D-dependent rickets, vitamin D-resistant rickets, immunological deficiencies like cyclic neutropenia, Chediak-Higashi syndrome, histiocytosis X, Papillon-Lefevre syndrome, leukocyte adhesion deficiency syndrome, and hypophosphatasia.^9^

We, hereby, present a case report on clinical manifestations of a 4%-year-old boy with a severe DI and its management using complete overlay dentures.

## CASE REPORT

**Fig. 1:**
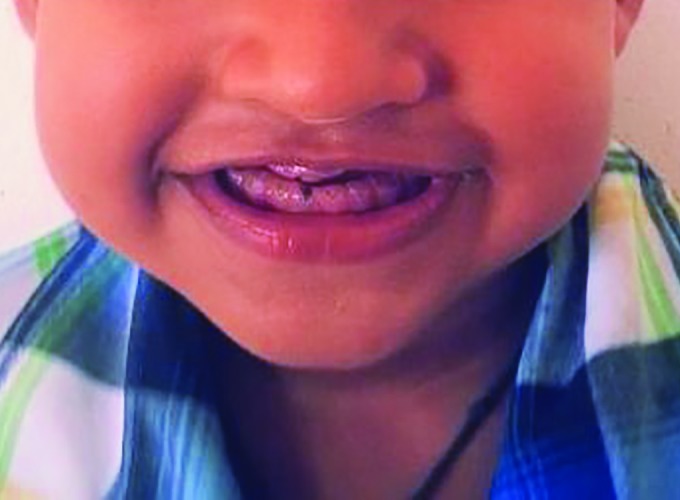
Pretreatment view of the patient with typical old man appearance

A 4%-year-old boy presented to the clinic complaining of constant peer harassment due to his “old man” appearance (Fig. 1). His mother also complained of bleeding from his upper right and left back teeth region.

History revealed that his primary dentition had an unsightly color deviation from the normal and continuous chipping of tooth structure. He never complained of pain or sensitivity in any of his teeth. A few days back, his mother noticed the presence of bleeding in relation to his upper back teeth while brushing. The boy was born after a full-term pregnancy and all milestones of development were normal. There was no history of unusual bone brittleness or any other systemic illness or drug usage in the present or past. The family history revealed that his mother is a diagnosed case of Shields DI type II, and she is wearing an overdenture for the past 6 years.

**Fig. 2:**
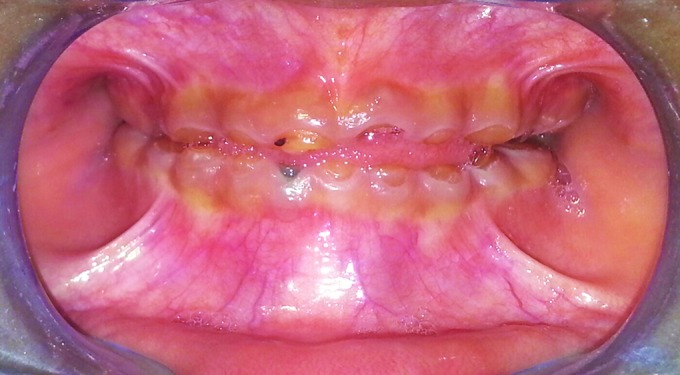
Pretreatment intraoral view with severely attrited teeth

On clinical examination, the child had typical appearance of an edentulous person with loss of vertical dimension, decreased lower facial height, prognathic facial profile, and loss of upper and lower lip support. On intraoral examination, all the deciduous teeth were attrited to the level of the gingiva. The 54, 64, 81, 51, and 61 presented as root stumps with mobility of 54 and 64. All the teeth were yellow-brown in color and translucent (Fig. 2).

**Fig. 3:**
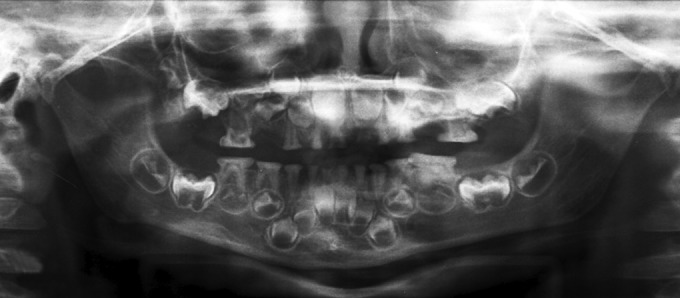
Orthopantomogram X-ray with severely attrited deciduous teeth with obliterated pulp chambers

### Radiographic Examination

**Fig. 4:**
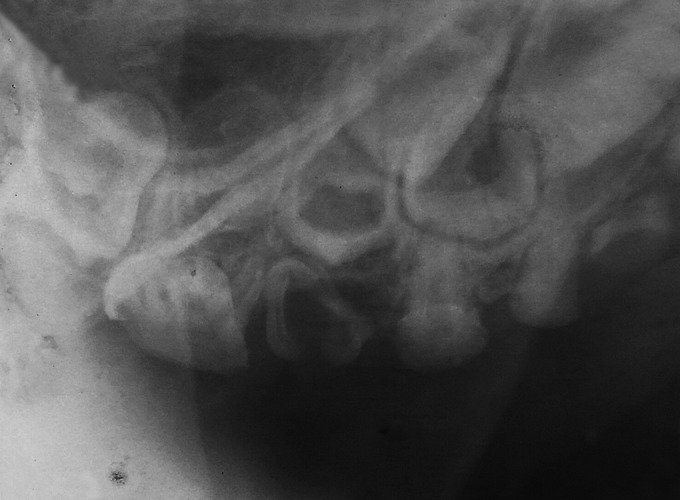
Intraoral periapical X-ray of upper right quadrant with marked cervical constriction and pulpal obliteration in deciduous teeth

The orthopantomogram (OPG) (Fig. 3) and intraoral periapical (IOPA) X-rays revealed bulbous crowns with marked cervical constriction of 55, 65, 74, 75, 84, 85. Total obliteration of pulp chambers and root canals of 52, 62,71,72,82 and partial obliteration of pulp chambers and root canals of all other teeth were also evident. The root stumps of 54, 64, 81, 51, and 61 showed resorption in the apical portion and periapical pathology (Fig. 4). The presence of developed permanent tooth buds was also evident. The child’s dental age determined radiographically was same as his chronological age.

After correlating the clinical and radiographic findings along with a positive family history, diagnosis of Shield’s DI type II was made. Since the crown structure of all teeth was severely attrited with inadequate tooth structure to support individual crowns, it was decided to fabricate an overlay denture for this patient. The treatment plan was explained to the patient’s mother and informed consent was obtained from her.

Voice control, distraction, tell-show-do technique, and positive reinforcement were the behavior-modification techniques employed for managing the patient.

Prior to denture fabrication, root stumps of 54, 51, 61, 64, and 81 were extracted under local anesthesia. Alginate impressions were made and study models made of stone plaster were surveyed to locate tooth undercuts that may prevent the denture from seating in proper position. Enameloplasty was performed on lingual aspect of 75 and 85 and buccal aspect of 55 and 65. Custom-made acrylic trays were made and border bolded, similar to an adult denture impression procedure. A final rubber base impression was taken and a cast was made of stone plaster.

Acrylic base plates with wax occlusal rims were made and the vertical dimension of occlusion was established. Due to the lack of alveolar ridge development and severe tooth wear, patient’s vertical dimension was considerably diminished. After recording the jaw relation, the casts were mounted on a hinge articulator.

**Fig. 5:**
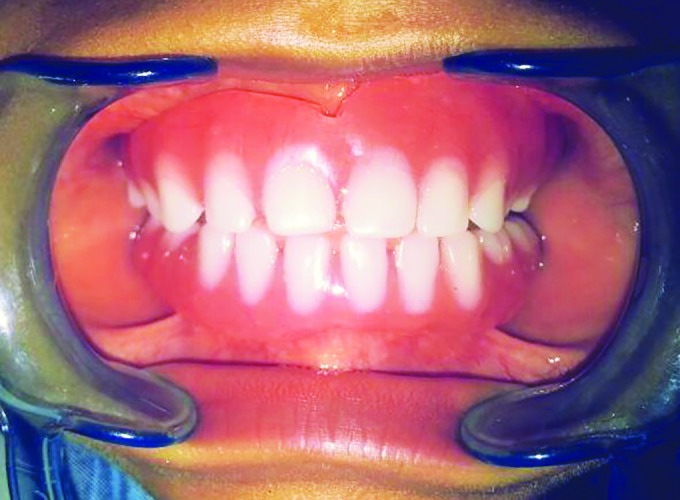
Denture seated in patient’s mouth

**Fig. 6:**
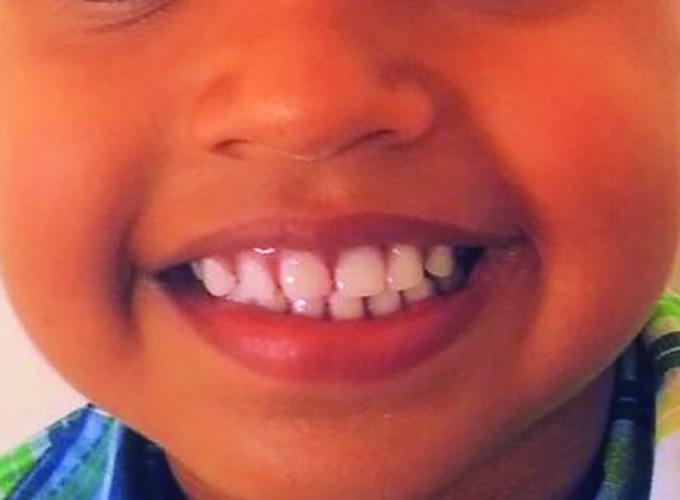
Posttreatment view with a confidently smiling patient

Teeth were selected after consultation with patient and parent. Smallest available permanent acrylic teeth were selected and teeth were recontoured to mimic deciduous teeth by grinding. To resemble natural deciduous dentition, teeth were arranged in wax with normal physiologic spacing and rounded free gingival margins. After a satisfactory try-in, the dentures were processed and then delivered to the patient after minor adjustments like relieving the upper labial frenum region (Fig. 5). The patient who was very reluctant to smile before treatment left the clinic with a confident beaming smile (Fig. 6).

Home care instructions were given, which included removal of the dentures at night and brushing both dentures and natural teeth after every meal. Since follow-up care is very important, patient is recalled on a regular basis for oral examination, dental prophylaxis, oral hygiene instructions, and denture adjustments, if necessary.

## DISCUSSION

The clinical severity of DI needs to be assessed when developing a treatment plan. The importance of esthetic looks cannot be ignored. The smile is very important for socialization and for the child’s self-concept from an early age.

The DI involves microscopic alterations in the morphology of dentin. One-third of the cases have associated defects in the calcification of enamel also (e.g., hypo-mineralizaion or hypoplasia). The primary dentition is more frequently damaged and subjected to rapid wear in comparison with permanent.

The objective of early treatment of DI should be to restore good occlusion and esthetics, favorable growth of facial bones and temperomandibular joints, favorable condition for eruption of permanent successors, prevention of loss of vertical dimensions, and restoration of vertical dimension, if already lost. Treatment may include prevention of caries, management of attrition, monitoring of the development of the craniofacial skeleton, placement of artificial crowns to prevent excessive loss of the tooth structure when deciduous teeth begins to wear, overdentures, and overlay dentures, if tooth structure is already extensively lost.^10^

Treatment strategies should be selected based on the age of the patient, severity of the problem, and the presenting complaint of the patient.

Management strategies for DI of primary dentition include

- Stainless steel crowns on posteriors helps to prevent tooth wear and maintain occlusal vertical dimension.
- Composite facing or composite strip crowns to improve esthetics.
- *Overdentures and overlay dentures:* If the patient is reporting late for treatment with teeth attrited till 9 10 gingiva.
  In mixed dentition cases
- The erupting permanents should be monitored for any increased wear
- Stainless steel crowns on permanent molars
- Composite covering of permanent anteriors
- Celluloid strip or polycarbonate crowns as an interim measure for permanent anteriors.^11^
  The DI of permanent dentition can be managed by
- Cast occlusal onlays: On first permanent molars and the premolars; help to minimize tooth wear and maintain occlusal vertical dimension.
- *Porcelain veneers or porcelain crowns:* to replace interim restorations when the child is old enough.
- Full-mouth rehabilitation with crowns and partial dentures.
- *Overdentures:* in cases with severe tooth wear.
- *Dental implants:* when growth is complete at about 18 years of age.^8^

In the present study, tooth structure was extensively lost. Two primary molars and three primary incisors had to be extracted because they presented as root stumps with mobility. Vertical dimension of occlusion had already collapsed. Patient’s primary concern was his edentulous “old man look.” Considering all this, it was decided to fabricate overlay dentures for this patient.

According to Brewer and Fenton, “An overlay denture is a complete or partial removable denture fabricated over retained teeth or roots that are not prepared with a coping to interface with the denture.”^10^

Lord and Teel state that “In over denture the remaining teeth needs endodontic treatment and cast metal copings.”^10^

Advantages of overlay dentures include

- Enhanced mastication and esthetics
- Alveolar bone is maintained by the retention of teeth
- Increased proprioception compared with complete denture because of retained teeth with their periodon-tal ligament
- In comparison with an overdenture overlay, denture is more economical, needs less chairside time, and does not require any specialized tooth preparation
- Has excellent retention and stability
- Positive psychological support to the child. Child may be saved from peer harassment resulting in increased self-esteem and confidence^10^

Regular follow-up care is most important. Patient should be recalled for dental prophylaxis, denture adjustments, and oral hygiene instructions. The dentures should be modified by adding acrylic as teeth exfoliate or erupting teeth should be accommodated by grinding concavities into the denture. Growth of the child may necessitate relining or sometimes remaking of the denture.^10^

## CONCLUSION

The DI is a developmental disturbance of teeth that is characterized by overproduction of dystrophic dentin. There is early chipping of enamel due to the absence of scalloping at the DEJ and decreased mineralization of dentin. Early diagnosis and intervention are needed for a favorable prognosis.

A multidisciplinary approach is essential for the preservation of the existing tissues and for restoration of function and esthetics. Prosthodontic rehabilitation as done in this case improves the function as well as esthetics and also provides psychological boosting for the affected patients.
